# 4-Phenylbutyrate Attenuates the ER Stress Response and Cyclic AMP Accumulation in DYT1 Dystonia Cell Models

**DOI:** 10.1371/journal.pone.0110086

**Published:** 2014-11-07

**Authors:** Jin A. Cho, Xuan Zhang, Gregory M. Miller, Wayne I. Lencer, Flavia C. Nery

**Affiliations:** 1 Division of Gastroenterology/Cell Biology, Boston Children's Hospital, Harvard Medical School, Boston, MA, United States of America; 2 Neuroscience Center, Department of Neurology, and Center for Molecular Imaging Research, Department of Radiology, Massachusetts General Hospital and Program in Neuroscience, Harvard Medical School, Boston, MA, United States of America; 3 Department of Pharmaceutical Sciences and Center for Drug Discovery, Northeastern University, Boston, MA, United States of America; 4 Harvard Digestive Diseases Center, Harvard Medical School, Boston, MA, United States of America; Louisiana State University Health Sciences Center, United States of America

## Abstract

Dystonia is a neurological disorder in which sustained muscle contractions induce twisting and repetitive movements or abnormal posturing. DYT1 early-onset primary dystonia is the most common form of hereditary dystonia and is caused by deletion of a glutamic acid residue (302/303) near the carboxyl-terminus of encoded torsinA. TorsinA is localized primarily within the contiguous lumen of the endoplasmic reticulum (ER) and nuclear envelope (NE), and is hypothesized to function as a molecular chaperone and an important regulator of the ER stress-signaling pathway, but how the mutation in torsinA causes disease remains unclear. Multiple lines of evidence suggest that the clinical symptoms of dystonia result from abnormalities in dopamine (DA) signaling, and possibly involving its down-stream effector adenylate cyclase that produces the second messenger cyclic adenosine-3′, 5′-monophosphate (cAMP). Here we find that mutation in torsinA induces ER stress, and inhibits the cyclic adenosine-3′, 5′-monophosphate (cAMP) response to the adenylate cyclase agonist forskolin. Both defective mechanins are corrected by the small molecule 4-phenylbutyrate (4-PBA) that alleviates ER stress. Our results link torsinA, the ER-stress-response, and cAMP-dependent signaling, and suggest 4-PBA could also be used in dystonia treatment. Other pharmacological agents known to modulate the cAMP cascade, and ER stress may also be therapeutic in dystonia patients and can be tested in the models described here, thus supplementing current efforts centered on the dopamine pathway.

## Introduction

The DYT1 early-onset primary dystonia is the most severe form of hereditary dystonia and is caused by a mutation in the gene, TOR1A (DYT1), encoding torsinA protein [1]. Current treatments include high dose anticholinergics, which are only partially effective and can compromise learning and memory [2]. Another available treatment for dystonia is deep brain stimulation (DBS), which involves an implant of stimulating electrodes into the basal ganglia [3]. Although DBS can be effective in treating some DYT1 dystonia patients, it is a neurosurgical procedure with concomitant risks and high cost. The DYT1 dystonia-associated protein, torsinA, is a ubiquitously expressed protein in many organs, including the central nervous system (CNS). Intriguingly, in patients with DYT1 dystonia, only the CNS is affected. The clinical phenotype of dystonia is thought to result from decreased function of torsinA causing defects in neuronal function, including abnormal dopaminergic neurotransmission in the basal ganglia [4]. However, the role of torsinA and the correlation between the dysfunction caused by the mutation and the dystonic phenotype remain unclear. Many studies at the biochemical, structural, and cell biological levels have been performed in order to characterize torsinA [5], [6]. These studies, together with the generation of several animal models [7]–[9], have contributed to the identification of cellular compartments and pathways affected by mutant torsinA, including the nuclear envelope (NE) [10], [11], cytoskeleton [12], [13], cell migration [12], [14], secretory pathway [15]–[18], dopamine pathway [5], [19]–[22], synaptic vesicle recycling [22], endoplasmic reticulum (ER) stress [23], [24], and endoplasmic reticulum-associated degradation (ERAD) [24], [25], where torsinA's function may be crucial for the cell homeostasis.

To define therapeutic pathways to target dystonia, it is important to identify cellular processes affected in the brain. Our studies with dystonia animal models and fibroblast cells from DYT1 patients have implicated decreased intracellular levels of cAMP in these DYT1 dystonia models when compared to controls. Alterations of the second messenger cAMP affect normal brain function such as in synapses, neuronal memory, and striatal plasticity [26]–[28], and can also be found in several neurological diseases, such as in Fragile X [29], [30], and in Huntington disease [31], [32]. In the cells, the concentration of cAMP is regulated by adenylate cyclases (AC) and phosphodiesterases (PDEs), respectively in its synthesis and its degradation [33].

Dopamine is the pathway most extensively studied in dystonia. Genetic mutations of the GCH1 gene, which codes for guanosine triphosphate cyclohydrolase I (GTPCH I) [34], the tyrosine hydroxylase (TH) gene [35], and the guanine nucleotide binding protein (GNAL) gene [36] are found in dystonia, and surprisingly share a common mechanism associated with the cyclic cAMP/PKA pathway. GCH1 and TH gene products participate in dopamine synthesis in the presynaptic neurons and both genes are regulated by cAMP [37]–[39]. GNAL gene encodes G α olf, a stimulatory subunit for heterotrimeric G-protein complexes (GPCR) [35]. Upon activation, G α olf stimulates the coupling of some GPCR subtypes such as dopamine receptor D1R, dopamine receptor D5DR, and adenosine receptor A2AR with adenylate cyclase, resulting in the generation of cAMP, and protein kinase A (PKA) activation [40].

Our previous studies suggest that the presence of torsinAΔE somehow contributes to ER stress load [23], [24]. We recently obseved that our DYT1 dystonia cell models have increase ER stress and reduced intracellular cAMP levels. While it is not clear how the mutant torsinAΔE affects ER stress and cAMP production, we find these defects are rescued by treatment with 4-phenylbutyrate (4- PBA), a small molecule that suppresses the ER stress response to misfolded proteins. The findings open new perspectives on repairing disrupted ER stress and cAMP signaling in dystonia, in which torsinA may play a new key role.

## Materials and Methods

### Ethics Statement

All human cell lines and protocols in the present study were carried out in accordance with the guidelines approved by institutional review boards (IRB) at Partners Human Research Committee (Protocol #: 2007P001632/MGH). Fibroblast cell lines received from Dr. Xandra Breakefield laboratory were previously collected through a Yale Medical School research protocol between 1974–1984. Individuals enrolled in this research protocol had skin biopsies taken, and signed consent forms. All animal study was carried out in strict accordance with the recommendations in the Guide for the Care and Use of Laboratory Animals of the National Institutes of Health. The protocol was approved by the Massachusetts General Hospital (MGH) Institutional Animal Care and Use Committee (IACUC) (Protocol Number: 2004N000271/MGH). All surgery was performed under anesthesia, and all efforts were made to minimize suffering.

### Animals

Heterozygous Tor1A knockout mice (Tor1A^+/−^) [9], and Heterozygous Tor1A knock-in mice (Tor1A^wt/ΔE^) [7], [9] were received from Dr. Yuqing Li (University of Florida, Gainesville, FL, USA). We crossed Tor1A^+/−^ and Tor1A^wt/ΔE^ mice to generate timed pregnancies. The day of vaginal plug detection was considered embryonic day 0 (E0). Embryonic neuron cultures were prepared from each embryo on embryonic day 15 (E15). The genotype of each embryo was identified as reported previously [7], [9].

### Mixed cortical and striatal neuron culture

Mixed cortical and striatal neuron cultures were prepared from invidual E15 mouse embryos. Briefly, embryos were removed by Caesarean section from a pregnant mouse. Cortices and striata from embryonic mice were dissected and stored in ice-cold 1 x phosphate buffered saline (PBS). The cortices and striata of individual embryos were dissociated with 0.625% trypsin at 37°C in neurobasal media (NBM, supplemented with B27, N2, 500 mg/ml streptomycin, 100 IU/ml penicillin and 2 mM L-glutamine, all from Invitrogen, Carlsbad, CA, USA). Trypsin was removed and Dulbecco's Modified Eagle Medium plus nutrient mix F12 (DMEM/F12, Invitrogen) supplemented with 10% fetal bovine serum (FBS, Invitrogen), N2 supplement and penicillin/streptomycin added to inactivate the trypsin. Cortices and striata were treated with 12.5 mg/ml DNase I (Sigma, St. Louis, MO, USA) in DMEM/F12+ FBS for 5 min at 37°C and homogenized. Cell suspension was then diluted in NBM supplemented with with B27, 0.5 mM L-glutamine. The cells were counted and then plated on a 96-well plate coated with polyethyleneimine (Sigma, St. Louis, MO, USA) in 0.15 M Borate Buffer at a density of 4×10^4^ cells/well. For each embryo, we generated approximately 8 wells. Half the media was replaced with fresh media every two days. The cells were treated with 5 µM cytosine arabinoside (ARA-C) four days after being plated and the total media was changed after 24 hours. The density of immunoreactive cells positive for βIII tubulin (a neuronal marker), and GFAP (a marker of astrocytes) was performed in parallel at 5–8 days in *vitro* (DIV). Results showed that the majority of cells (99.5%) were positive only for βIII tubulin. Note that cultures from individual neurons (an average of 10 well in 96 plates per embryos) were maintained for 10 days, and were then used for cAMP assay.

### Mouse embryonic fibroblast cells (MEFs)

MEF cultures were prepared as described [12], [41] from single embryos (embryonic day 15) of mating between heterozygous torsinA knockout mice or torsinA knock-in mice. Cells were grown in DMEM (GIBCO, Rockville, MD) as described [41]. MEFs cells were seeded, and incubated overnight before the cAMP assay.

### Human fibroblast cell culture

Primary human skin fibroblasts of control fibroblasts (HF6, HF19) and of DYT1 patient's fibroblast (HF48) were generated in Dr. Breakefield ‘s laboratory as described previously [42], under IRB approved guidelines. Fibroblast control cell lines GM00023, GM00024, GM01651, GM02912, and DYT1 patient fibroblast cell lines GM02551, GM03208, GM032011, GM02306 were purchased from Coriell Institute for Medical Research (Camden, NJ). The Coriell Cell Repository maintains the consent and privacy of the donor fibroblast samples. DYT1 patient fibroblast cells, FFF13111983 [43], were provided from Dr. Mirella Filocamo (Biobank from Patients affected by Genetic Diseases, L'Istituto Giannina Gaslini, Genoa, Italy), a member of the Telethon Network of Genetic Biobanks (project no. GTB12001). The primary fibroblast cells were grown in culture, as described [24]. For more information about the human fibroblast cell lines, see Table S1. Human fibroblast cells were seeded, and incubated overnight before the cAMP assay.

### Western blot

SDS-PAGE electrophoresis and protein transfer were carried out as described [23]. Membranes were probed with antibodies against β-actin (1∶5,000), and mouse torsinA (1∶1000) (Abcam) diluted in TBST and visualized with HRP conjugated to secondary antibodies and SuperSignal West Pico Chemiluminescent Substrate (Pierce). Secondary antibodies for Western blots were sheep anti-mouse IgG-HRP (1∶10,000) or donkey anti-rabbit IgG-HRP (1∶10,000) (Amersham Pharmacia Biotech, Piscataway, NJ).

### Chemicals

Forskolin (Sigma, St. Louis, MO, USA) was dissolved in DMSO at 10 mM concentration. Thapsigargin (Sigma, St. Louis, MO, USA) was suspended in DMSO at 3 mM. The phenylbutyric acid or 4-phenylbutyrate (4-PBA; Sigma, St. Louis, MO, USA) was dissolved in filtered sterile water at a 1 M stock concentration.

### cAMP ELISA Assay

We have optimized an ELISA cAMP assay (GE Healthcare) to determine cAMP intracellular levels in dystonia cell models. Briefly, neuron cells were seeded in a 4×10^4^ cells/well (neurons) and 5×10^3^ cells/well (fibroblasts) in 96-well plates. Fibrolast cells were incubated overnight before the assay, and neurons were culture for 10 days prior to the assay. After 30 minutes incubation with or without 10 µM forskolin, the cAMP levels were determined according to the manufacture's instructions. At least three independent experiments were performed for each assay. Data were normalized to the amount of protein in each sample, as determined using a dye-binding protein assay (Bio-Rad).

### ATP/Cell viability analysis

Neuronal and fibroblast viability was evaluated by CellTiter-Glo luminescent cell viability assay (Promega, Madison, WI) to determine the number of viable cells in culture based on the quantitation of ATP levels, indicating the presence of metabolically active cells. Briefly, primary neurons and fibroblast cells were grown as described above and a volume of CellTiter-Glo Reagent was added to each well equal to the volume of cell culture medium. Then, the contents were mixed for 2 min on a shaker to induce cell lysis and the plates were incubated at room temperature for 10 min in the dark. Cellular luminescence intensity was measured using a Luminometer (Dynex Technologies, Chantilly, VA).

### Quantitative real-time qPCR

Total RNA of cells was extracted with RNeasy mini kit (Qiagen, Valencia, CA). The cDNA was prepared from total RNA using Superscript first-strand synthesis system (Invitrogen) with the oligo (dT)^12–18^ primers, according to the manufacturer's instructions. RNA samples were treated with DNase I (Invitrogen) for the elimination of genomic DNA. Human spliced form of XBP1 mRNA expression (forward: 5′-AACCAGGAGTTAAGACAGCGCTT-3′, reverse: 5′-CTGCACCCTCTGCGGACT-3′) was determined by real-time qPCR using relative quantitation by the comparative threshold cycle number (Ct) method, iCycler, and SYBR Green Ready-mix (Bio-Rad, Hercules, CA) and normalized by glyraldehyde-3-phosphate dehydrogenase (GAPDH) expression. Preliminary experiments were performed with each primer pair to determine the amplification temperature that provided an optimal correlation between template concentration and signal intensity. At least three independent experiments were performed for each assay.

### Data analysis and statistics

Statistical analysis was performed using GraphPad Prism software (version 3.0). Results are expressed as mean ± S.E.M. of at least three separate experiments unless otherwise indicated. Statistical significance of comparison between two groups was determined by two-tailed Student's t-test where indicated. Multiple comparison analyses of variance between the groups were performed by a one-way ANOVA test, followed by a Tukey's multiple comparison post hoc tests. Significant differences were considered at p values of less than 0.05.

## Results

### Abnormal torsinA function downregulates cAMP production in dystonia mice and human cell models

We first observed a potential relationship between mutant torsinA and adenylate cyclase during our studies using cholera toxin to examine the role torsinA might play in ERAD [24]. We found that cAMP levels in cell lines expressing mutant torsinA were lower after treatment with cholera toxin, a well known cAMP agonist, when compared to control cell lines.

**Figure 1:**
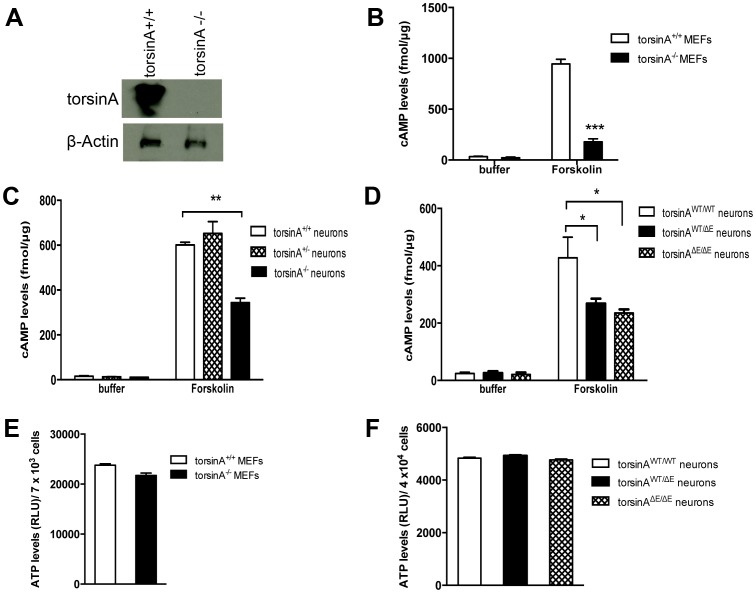
Absence of torsinA or presence of torsinAΔE, downregulates cAMP production in dystonia animal models. (A) Lysates from mouse embryonic fibroblasts (MEFs) cells from controls (torsinA^+/+^) and MEFs from knockout torsinA mice (torsinA^-/-^) were resolved by SDS-PAGE, and immunoblotted with antibodies specific to mouse torsinA (torsinA), and β-Actin, showing the absence of torsinA in torsinA^-/-^ MEFs. (B) To analyze differences in cAMP levels in torsinA^+/+^, and torsinA^-/-^ MEFs, the cAMP production was measured under basal conditions and in response to the direct adenylate cyclase agonist forskolin (10 µM). Although no significant differences in basal cAMP production were observed in MEFs cells, there was a 5-fold reduction (***P<0.001; Student t-test, n = 3 experiments) in torsinA^-/-^ MEFs after forskolin stimulation compared to control torsinA^+/+^ MEFs. (C) Primary mixed cortical and striatal neuronal cultures of individual E15 embryos from controls littermates (torsinA^+/+^), heterozigous Knockout torsinA (torsinA^+/-^), and homozigous Knockout torsinA (torsinA^-/-^). No signicant differences in basal cAMP production were observed, however a 1.5 fold reduction of cAMP levels was also observed in torsinA^-/-^ neurons (**P<0.01; Student t-test, n = 3 experiments) after forskolin stimulation compared to control torsinA^+/+^, neurons. (D) Same results were found in knock-in dystonia models. A 1.2 fold reduction in the cAMP production was obseved in heterozygotes Knock-in (torsinA^WT/ΔE^) neurons (*P<0.05; one-way ANOVA and Tukey's post hoc comparison, n = 3 experiments), and a 1.4 fold reduction in homozygotes Knock-in (torsinA^ΔE/ΔE^) neurons (*P<0.05; one-way ANOVA and Tukey's post hoc comparison, n = 3 experiments) in forskolin-stimulated cAMP production cells compared to control torsinA^wt/wt^ neurons. (D) Since adenylate cyclase catalyzes the conversion of ATP to cAMP, intracellular ATP levels were measured from torsinA^-/-^ MEFs and control torsinA^+/+^ MEFs (E), and in heterozygous or homozygous Knock-in neurons (F), when compared to controls. The intracellular ATP levels were determined from cell lysates using the ATP-Glo™ Bioluminometric Cell Viability Assay. No significant differences were found in the ATP levels (n = 3 experiments).

Thus, to test the hypothesis that torsinA status might affect agonist-induced adenylate cyclase activities, we examined cAMP production in several dystonia models using forskolin, which is a general and direct activator of the adenylate cyclases. We first demonstrated complete lack of torsinA expression in torsinA knockout model (Figure 1A). Then, we investigated the forskolin-induced cAMP response in wild-type (torsinA^+/+^), and homozygous (torsinA^-/-^) MEFs (Figure 1B), as well as from torsinA^+/+^, torsinA^+/-^, and torsinA^-/-^ neurons from knockout dystonia mice (Figure 1C). Results showed a strong loss of cAMP signal in torsinA^-/-^ MEFS (n = 3; p<0.001; Figure 1B), and in torsinA^-/-^ neurons (n = 3; p<0.01; Figure 1C) compared to wild-type controls upon stimulation of the cells with forskolin. Similar results were obtained in primary neuron cultures from torsinA knock-in mice (Figure 1D). The presence of torsinAΔE in heterozygous and homozygous neurons led to lower levels of cAMP (n = 3; p<0.05; Figure 1D), when compared to controls. Furthermore, we confirmed that the inhibition of forskolin-induced cAMP production in cells lacking torsinA or expressing mutant torsinA, torsinAΔE, were not due to a reduction of intracellular levels of ATP in our DYT1 dystonia models, which could have confounded our results (Figure 1E, and 1F). There were no significant differences in the ATP levels measured in this study between wild type cells and cells from knockout (Figure 1E) or knock-in mice (Figure 1F).

**Figure 2:**
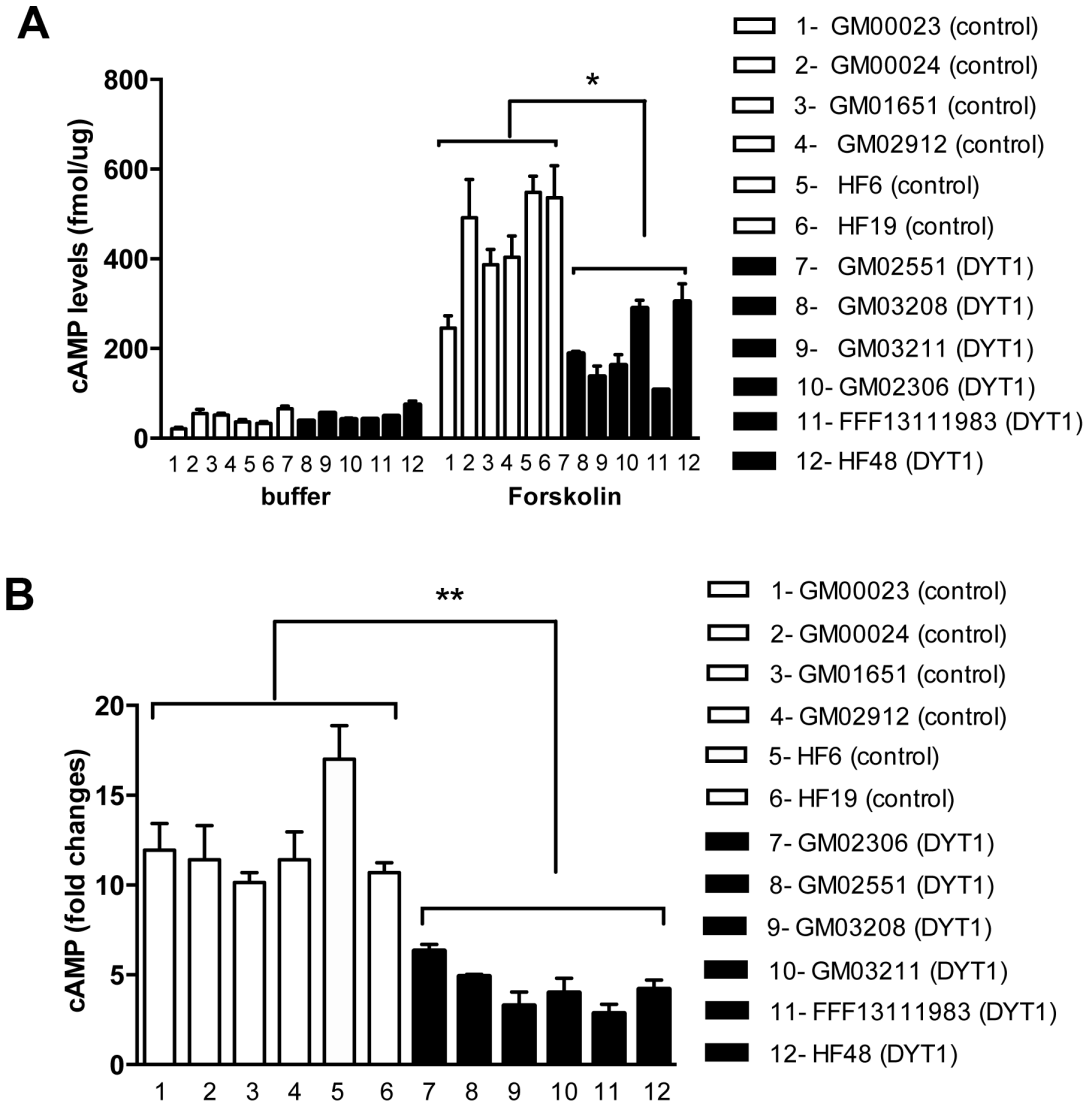
Adenylate Cyclase (AC) activity is reduced in DYT1 dystonia patient fibroblast cells. (A) A 2.5-fold reduction (*P<0.05; one-way ANOVA and Tukey's post hoc comparison, n = 3 experiments) in the cAMP accumulation was observed by DYT1 patient fibroblast cell lines (6 cell lines represented in black 7–12: GM02306, GM02551, GM3208, GM03211, FFF13111983, HF48) compared to healthy fibroblast cell lines (6 cell lines represented in white 1–6: GM00023, GM00024, GM01651, GM02902, HF6, HF19) in response to forskolin (10 µM). The line GM00023 from a healthy subject (1) showed no significant increase when compared to DYT1 patient fibroblast lines. (B) Results are also expressed as fold increase over basal cAMP level of each cell line. All DYT1 fibroblast lines have decreased response to forskolin stimulation compared to healthy fibroblast cells, including GM00023 (**P<0.01; one-way ANOVA and Tukey's post hoc comparison, n = 3 experiments).

To test whether these results translate to human cell models, we investigated whether cAMP levels were also altered in human fibroblast lines prepared from various dystonia DYT1 patients and healthy controls. At rest, there was no overall difference between control and DYT1 fibroblasts in basal levels of intracellular cAMP (Figure 2A). After forskolin stimulation, however, the induction of cAMP levels in DYT1 cells was significantly lower compared to control cells (n = 3; p<0.05; Figure 2A). One of the control cell lines, GM00023B, presented the lowest cAMP response to forskolin among control cell lines, and comparable in magnitude to the signals observed in DYT1 fibroblasts. However, we also observed that the GM00023B cells also presented the lowest basal level of cAMP, suggesting a general lower activity of adenylate cyclase in this specific line. Thus, to control for this, the cAMP levels induced by forskolin were normalized to the basal level of cAMP for each cell line and represented again by fold change after forskolin treatment. When the data was normalized to cell basal levels, all fibroblasts from healthy patients had uniformly increased responses to forskolin stimulation compared to all DYT1 fibroblast lines (n = 3; p<0.001; Figure 2B).

### 4-PBA, an ER stress inhibitor, rescues ER stress and cAMP defects in DYT1 patient fibroblasts

**Figure 3:**
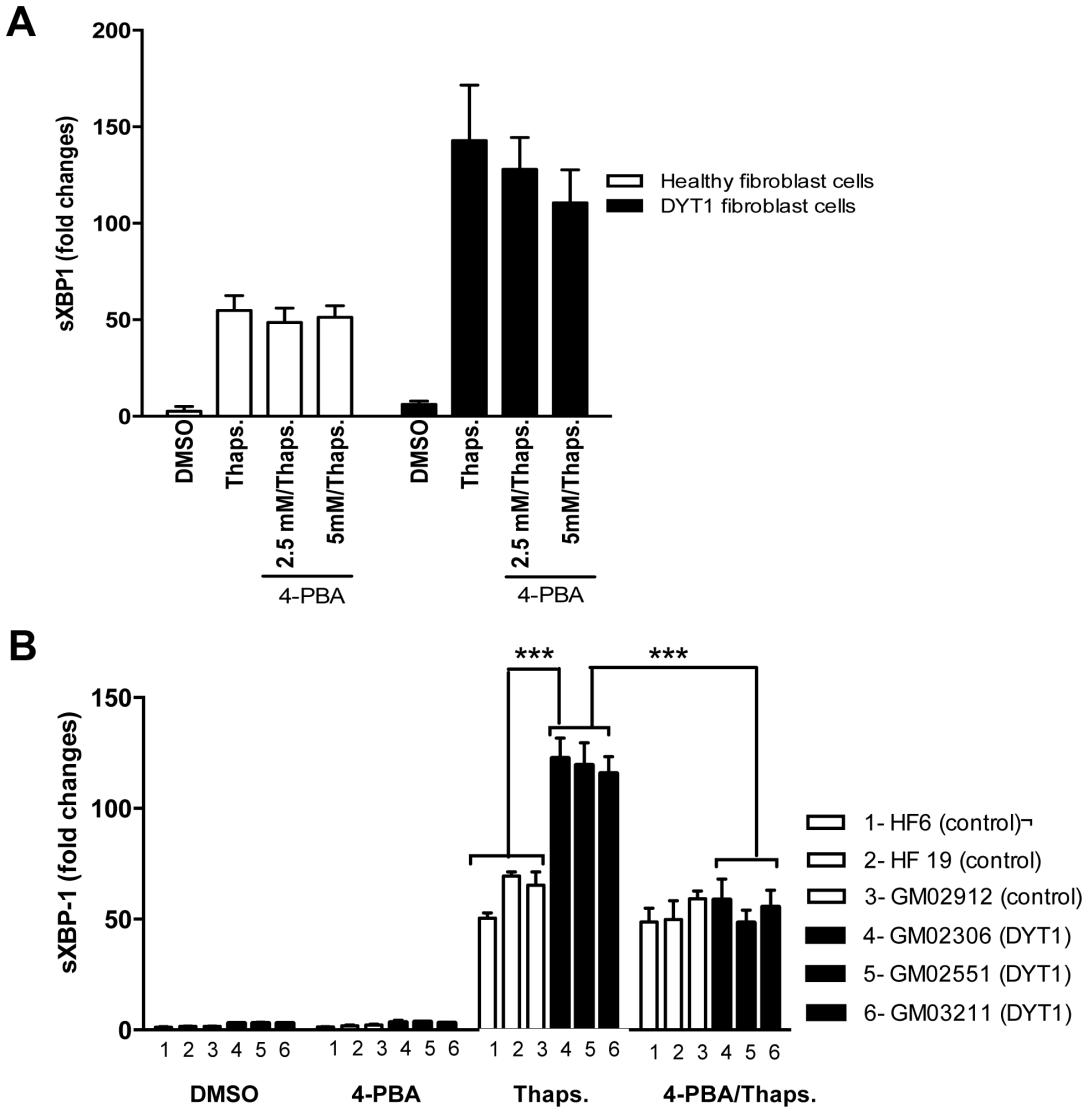
4-PBA alleviates ER stress in dystonia models. (A) Healthy control fibroblast cells lines (average of 3 lines: HF6, HF19 and GM02912) and DYT1 patient fibroblast lines (average of three lines: GM02306, GM02551 and GM03211) were treated for 18 hours in the absence or presence of 2.5 or 5 mM concentrations of 4-PBA, then challenged or not with 1 µM thapsigargin (Thaps.) for 3 hours. We have not observed significant differences in sliced XBP1 (sXBP1) levels, an ER stress marker, upon 4-PBA treatments (2.5 and 5 mM) in healthy control or DYT1 fibroblast cells. (B) As shown in (A), healthy fibroblast cells lines (3 lines represented in white 1–3: HF6, HF19, and GM02912) and DYT1 patient fibroblast cell lines (3 lines represented in black 4–6: GM02306, GM02551, and GM03211) were treated for 18 hours in the absence or presence of 10 mM concentration of 4-PBA, then challenged or not with 1 µM thapsigargin (Thaps.) for 3 hours. DYT1 fibroblasts cell lines have increased levels of mRNA expression levels of sliced XBP1 (sXBP1), an ER stress marker, compared to healthy controls (**P<0.01; one-way ANOVA and Tukey's post hoc comparison, n = 3 experiments) when stimulated with thapsigargin. However, when DYT1 fibroblast cells were pre-treated with 10 mM 4-PBA, they showed reduced sXBP1 levels, similar to healthy control lines. A 1.2 reduction in the sXBP levels was observed when DYT1 fibroblast cells challenged with thapsigargin, and treated with 4-PBA (**P<0.01; one-way ANOVA and Tukey's post hoc comparison, n = 3 experiments). The results indicate that the presence of torsinAΔE induces ER stress in fibroblast cells, however, 10 mM 4-PBA treatment decreases ER stress in DYT1 patient lines.

We previously reported that the absence of torsinA or the presence of malfunctioned torsinAΔE induces ER stress [23], [24]. Here, we confirmed the activation of ER stress in DYT1 patient fibroblast cells by quantitative real-time PCR (qPCR) of the spliced form of the transcription factor X-box binding protein 1 (sXBP1). Our results show that DYT1 fibroblasts induced more XBP1 splicing (Figure 3) in both unstimulated (DMSO treatment), and stimulated (Thapsigargin treatment) conditions compared to control cells. Also we confirmed that neurons expressing torsinAΔE have higher levels of sXBP1 compared to control neurons (Figure S1) in unstimulated conditions. This suggests that the presence of torsinAΔE enhances ER stress.

Furthermore, we investigated whether 4-PBA, a small molecule with chaperone-like activity, may decrease ER stress in our dystonia cell models. 4-PBA major function is to scavenge ammonia and glutamine [44], however, recent studies have described 4-PBA as a potent inhibitor of HDAC [45], and function as a chemical chaperone during ER stress [46]. Rubenstein et al. first discovered the chaperone-like activity of 4-PBA when examining its effect on the trafficking of CFTRΔF508 to the cell surface [47]. In ER stress, 4-PBA may improve mislocalization, and/or aggregation, and trafficking of proteins associated with human disease [48].

**Figure 4:**
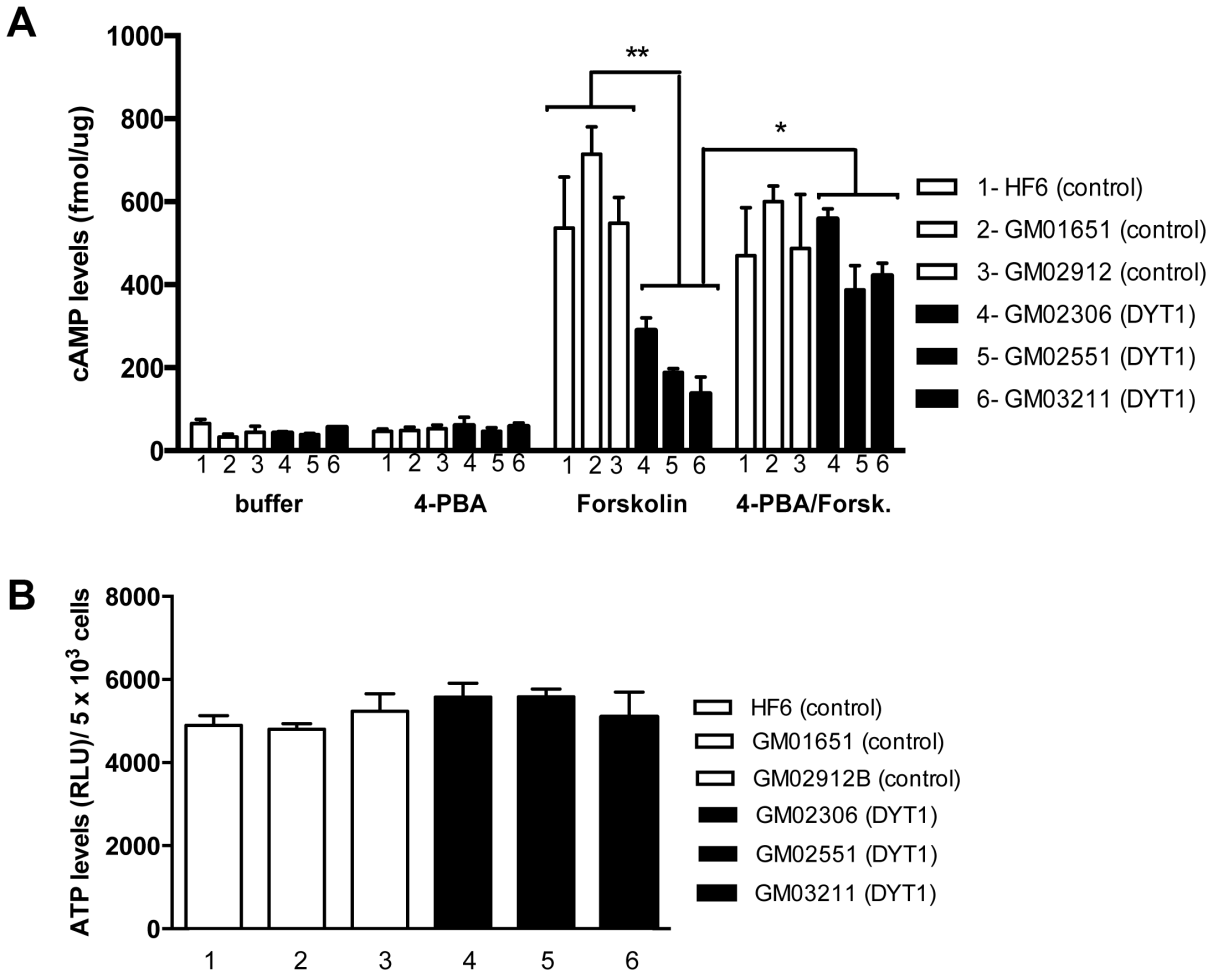
cAMP defects in dystonia models are rescued by 4-PBA treatments. (A) Healthy control fibroblast cells (3 lines represented in white 1–3: HF6, GM01651, and GM02912) and DYT1 patient fibroblast cells (3 lines represented in black 4–5: GM02306, GM02551, and GM03211) were pre-treated with or without 4-PBA for 18 hours, followed by 10 µM forskolin stimulation for 30 minutes. As observed previously, DYT1 fibroblast lines have significantly lower levels of cAMP accumulation after forskolin stimulation compared to healthy control fibroblast lines (**P<0.01; one-way ANOVA and Tukey's post hoc comparison, n = 3 experiments). However, the 4-PBA treatments also significantly rescued cAMP accumulation deficiency in DYT1 patient fibroblast lines comparable to control fibroblasts cAMP levels (*P<0.5; one-way ANOVA and Tukey's post hoc comparison, n = 3 experiments). (B) No significant changes were observed in the intracellular ATP levels from healthy control cells (HF6, GM01651, GM02912) and DYT1 fibroblast cells (GM2306, GM02551, GM3211).

In parallel experiments, gradual titrations of 4-PBA (2.5, 5, 10, 15 mM, 20 mM) were performed to assess the effects of PBA on ER stress in health fibroblast cells, and in DYT1 fibroblast cells. We observed, however, that treatment with concentrations 2.5 and 5 mM did not affect ER stress in health fibroblast cells, or in DYT1 fibroblast cells (Figure 3A). However, the treatment with 10 mM 4-PBA abrogated the thapsigargin response in DYT1 patient fibroblast cells (n = 3; p<0.001; Figure 3B), but had no effect on the response to thapsigargin in healthy fibroblast cells. In contrast, higher concentrations 15 and 20 mM 4-PBA were toxic for both cell types (data not shown). This result suggests that reduced chaperone function in cells expressing torsinAΔE may be responsible for increased ER stress. Since impaired expression of G protein coupled receptors (GPCRs) may be responsible for deficits in the cAMP/PKA signaling, we assessed whether mitigation of ER stress with 4-PBA could rescue the cAMP defects of DYT1 fibroblast cells (Figure 4A). We observed that the treatment with 4-PBA enhanced the cAMP response to forskolin in DYT1 patient fibroblast cells, with signals comparable to control cell lines (n = 3; p<0.05; Figure 4A). We note that pretreatment of 4-PBA reducing ER stress did not affect the forskolin-stimulated cAMP level in the control cell lines (Figure 4A), suggesting that 4-PBA had effects only on cells expressing mutant torsinA. Thus, 4-PBA appears to rescue both ER stress and cAMP accumulation in DYT1 fibroblast cells. In addition, we observed no significant changes in ATP levels measured in this study between healthy fibroblast cells and DYT1 patient fibroblast cells (Figure 4B).

## Discussion

**Figure 5:**
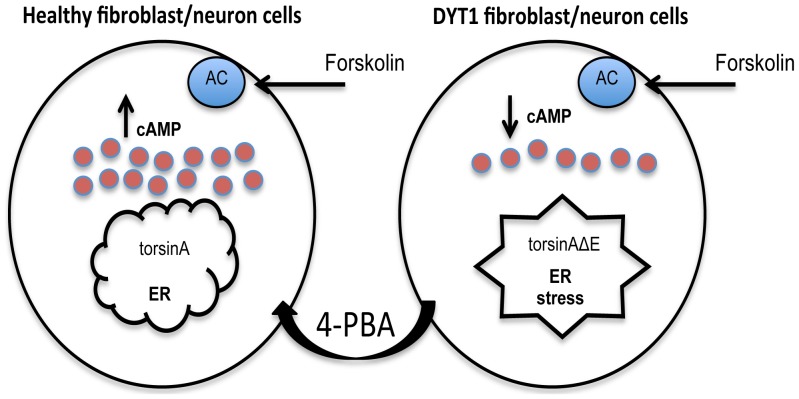
Model of torsinA modulation of cAMP. In healthy fibroblast cells (left panel), forskolin stimulates adenylate cyclase (AC) to synthesis cAMP, and there is no presence of Endoplasmic Reticulum (ER) stress in normal conditions. In absence of torsinA or in presence of mutant torsinA, torsinAΔE (right panel), cells present increased ER stress and lower cAMP accumulation. However, 4-PBA treatment rescues both ER stress and cAMP accumulation defects in DYT1 dystonia cell models by unoknow mechanism (s).

Here, we addressed the involvement of torsinA in the response to ER stress and to the cAMP pathways. How these two pathways are linked mechanistically remains unclear, but it is possible though that the ER stress induced by mutant torsinA in patients with DYT1 dystonia contributes to neuronal dysfunction through a cAMP defect (Figure 5). In our previous studies on the torsinA-related AAA-ATPase p97, which also operates in ERAD and induces ER stress upon dysfunction, we found an inhibitory effect on adenylate cyclase when p97 was mutated [49]. Remarkably, we also observed that 4-PBA treatments can attenuate the ER stress caused by torsinA mutants and this is associated with rescue of the forskolin-induced cAMP-response. We propose that 4-PBA might have therapeutic benefits in patients with dystonia.

Defective proteasome function and ER stress have a contributing role in various neurologic diseases [50]. In response to stress, cells temporarily decrease protein synthesis, delay membrane trafficking, increase movement of unfolded proteins out of the ER into the cytoplasm via ERAD, and up-regulate transcription of some chaperone proteins [51]. Recently, our group has shown an aberrant regulation of proteasome activity in dystonia [24]. Protein components of the ERAD pathway, for example, p97 and Derlin-1, as well as torsinA, have been found in inclusion bodies in brain and peripheral nerves from patients with a variety of neurodegenerative diseases, as well as dystonia [52]–[55]. Additionally, complementary evidence indicates that torsinA can reduce ER stress caused by ER protein overload in nematodes, and that torsinA deficient MEFs have increased levels of ER stress, as compared with wild-type MEFs [23], [24]. Interestingly, it has been proposed that ER stress can cause down-regulation of cAMP production in diabetes [56], and that a cAMP elevating reagent (CGS21680, an adenosine A2 receptor agonist) enhances proteasome activity in synapses of the striatum of R6/2 Huntington mouse model [57]. Interestingly, it has been shown that the proteasome activity can be regulated by PKA [58]. Future studies are needed to determine whether the impairment of proteasome activity in cells expressing torsinAΔE may cause accumulation of PKA regulatory subunits and/or suppression of PKA activity.

Our findings linking ER stress and cAMP signaling in dystonia models, also highlights that there is much to consider about striatal signaling to cAMP and PKA. Benzodiazepines are frequently used in the treatment of dystonia. This drug has muscle-relaxant properties and alleviates pain caused by muscle spasms and various dystonias [59], [60]. Interestingly, the administration of diazepam, a benzodiazepine anxiolytic, in rhesus monkeys, is known to increase cAMP by inhibiting PDE cAMP hydrolysis [61].

Numerous lines of evidence have linked dopaminergic dysfunction to dystonia [4], [5]. The physiological actions of dopamine are mediated by five distinct but closely related G protein-coupled receptors (GPCRs) that are divided into two major groups: the D1 (D1, D5) and D2 (D2, D3. D4) classes of dopamine receptors [62]. The D2 class is coupled to inhibitory G-proteins (G0/i) that decrease adenylate cyclase activity and cAMP levels, in contrast to the D1 class which acts through stimulatory G-proteins (e.g. Gsα) to increase activity and cAMP levels [63]. Dystonia has been associated with mutations in genes encoding proteins critical for dopamine (DA) signaling, such as GTP-cyclohydrolase [34], tyrosine hydroxylase [35], as well as polymorphisms in the DA receptor subtype, D5R [64], and GNAL dystonia gene, which encodes G α olf, a central component for striatal responses to dopamine (DA) [36]. Furthermore, several imaging studies showed various DA-related abnormalities, such as altered receptor binding in basal ganglia, in patients with different forms of dystonia [65]–[67]. Interestingly, it has been shown that adenylate cyclase activation by forskolin is highly potentiated in the presence of stimulatory G-proteins, such as G α olf [68]. Mutations in GNAL remove essential functional regions of Gαolf, responsible for activation of the G protein receptor D1 and Adenosine Receptor A2 (A2R), affecting cAMP accumulation. Thus, it is possible that mutations in GNAL and TOR1A genes may lead to dystonia via a common mechanism related to cAMP signaling. Perhaps torsinA participates in the proper folding/oligomerization of GPCR, or stimulatory G-proteins, reducing the function of these proteins.

Our results suggest that 4-PBA may have clinical utility in the treatment of dystonia. 4-PBA is a low molecular weight fatty acid already approved for clinical use as an ammonia scavenger in children with urea cycle disorders [69]. Several groups revealed that 4-PBA reverses the mislocalization and/or aggregation of the protein associated with human disease, including the ΔF508 cystic fibrosis transmembrane conductance regulator [47], mutant α1-antitrypsin [70]. In addition, 4-PBA improves the trafficking of the serotonin transporter (SERT) [71], by reversing the misfolded proteins and decreasing the protein folding load in the ER, therefore providing protective effects against ER stress. A previous study has shown that 4-PBA treatment decreases ER stress with regard to the following general ER stress markers: BiP, CHOP, activating transcription factor 4 (ATF4), as well as sXBP1 [56]. Additionally, 4-PBA alleviates ER stress by the Pael receptor, which is thought to be accumulated in PARK2, one type of inherited Parkinson's disease [72], reduced the area of damage due to ischemia in a rat model of cerebral infarction [73], it has been found to prolong life [74], and contribute to therapy for spinal muscular atrophy [75] by altering the pattern of gene expression.

4-PBA has high potential to treat neurologic diseases because it penetrates the blood–brain barrier, and exhibits significant neuroprotective effects in mouse models of neurodegenerative diseases, such as Alzheimer's disease (AD) [76], and Parkinson's disease (PD) [77]. As described above 4-PBA has multiple applications. It can be explained by the fact that 4-PBA has also multiple functions. First, 4-PBA may act as a chemical chaperone preventing the mislocalization and/or aggregation of proteins associated with human disease. Second, 4-PBA also possesses histone deacetylase (HDAC)-inhibitor activity. Initially HDAC inhibitors were designed as anti-cancer agents because of their important roles in the epigenetic regulation of protein translation. Recently, HDAC inhibitors have been implicated in the treatment of many neurodegenerative diseases [78]. Furthermore, the HDAC inhibitory function of 4-PBA has also been studied in neurodegenerative diseases related to abnormal histone acetylation, such as Huntington's disease (HD) [79], amyotrophic lateral sclerosis (ALS) [80], and spinal muscular atrophy (SMA) [75].

The precise steps by which mutant torsinA leads to impaired cAMP accumulation are not known, but it may involve an ER dysfunction. Further studies are needed to test this hypothesis, and to determine the efficacy of treatment of DYT1 dystonia that targets both ER stress and cAMP cascade. More studies are also needed to elucidate whether 4-PBA is functioning as a HDAC inhibitor or as a molecular chaperone in our dystonia cell models. This will provide insightful information for the structural optimization of this compound. Nevertheless, therapies working through the ER stress and cAMP cascade in the striatum may have the potential to benefit dystonia patients by alleviating dystonic symptoms. Animal studies will determine whether 4-PBA can be used to treat, as well as prevent, DYT1 dystonia.

## Supporting Information

Figure S1 Neurons expressing torsinAΔE has higher ER stress compared to control neurons. Total mRNA was prepared from a mixed neuronal culture of dissociated primary cortical and striatal at E15 from controls littermates torsinA^wt/wt^, and heterozygotes torsinA^wt/ΔE^ knock-in dystonia mice (*P<0.001; Student t-test, 2 independent experiments, with average of 2–3 embryos per genotype). Relative expression of sliced sXBP1 mRNA levels was measured by quantitative PCR. Results might indicate that the presence of torsinAΔE increases ER stress in neurons.
(PDF)

Table S1 Human fibroblast cells used in this study.
(PDF)
