# Supplementary material for: 4-Phenylbutyrate Attenuates the ER Stress Response and Cyclic AMP Accumulation in DYT1 Dystonia Cell Models
Source: PLoS One. 2014 Nov 7;9(11):e110086. doi: 10.1371/journal.pone.0110086 (PMC4224384; doi:10.1371/journal.pone.0110086)
Supplement: Figure S1 — Neurons expressing torsinAΔE has higher ER stress compared to control neurons. Total mRNA was prepared from a mixed neuronal culture of dissociated primary cortical and striatal at E15 from controls littermates torsinAwt/wt, and heterozygotes torsinAwt/ΔE knock-in dystonia mice (*P<0.001; Student t-test, 2 independent experiments, with average of 2–3 embryos per genotype). Relative expression of sliced sXBP1 mRNA levels was measured by quantitative PCR. Results might indicate that the presence of torsinAΔE increases ER stress in neurons. (PDF) [file pone.0110086.s001.pdf]

Supporting Information

Supplementary Figure S1.

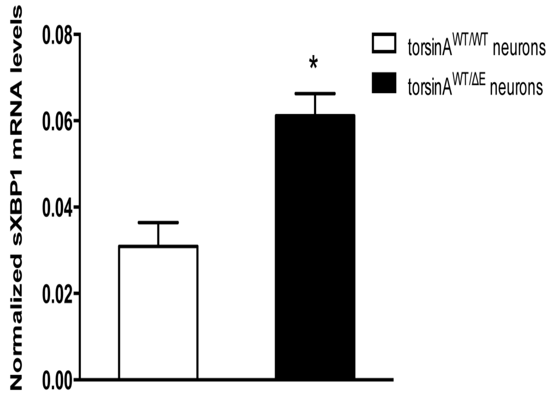

Supplementary Figure 1.

Neurons expressing torsinAΔE has higher ER stress compared to control neurons.

Total mRNA was prepared from a mixed neuronal culture of dissociated primary cortical and striatal at E15 from controls littermates torsinA$^{wt/wt}$, and heterozygotes torsinA$^{wt/\Delta E}$ knock-in dystonia mice (**P*<0.001; Student t-test, 2 independent experiments, with average of 2-3 embryos per genotype). Relative expression of sliced sXBP1 mRNA levels was measured by quantitative PCR. Results might indicate that the presence of torsinAΔE increases ER stress in neurons.
