# Supplementary material for: 4-Phenylbutyrate Attenuates the ER Stress Response and Cyclic AMP Accumulation in DYT1 Dystonia Cell Models
Source: PLoS One. 2014 Nov 7;9(11):e110086. doi: 10.1371/journal.pone.0110086 (PMC4224384; doi:10.1371/journal.pone.0110086)
Supplement: Table S1 — Human fibroblast cells used in this study. (PDF) [file pone.0110086.s002.pdf]

Supplementary Table S1.

Human fibroblast cells used in this study.

Cell Line	Genotype	Source	Gender	Age (YR)	Initial passage	Passage used
GM00023	Healthy	Coriell	Female	31	8	10-20
GM00024	Healthy	Coriell	Male	31	8	10-20
GM01651	Healthy	Coriell	Female	13	12	14-20
GM02912	Healthy	Coriell	Male	29	9	11-20
HF6	Healthy	Breakefield	nd	22	7	9-20
HF19	Healthy	Breakefield	Female	27	2	8-20
GM02306	DYT1	Coriell	Male	13	3	8-20
GM02551	DYT1	Coriell	Female	31	3	8-20
GM03208	DYT1	Coriell	Male	29	2	8-20
GM03211	DYT1	Coriell	Male	39	2	8-20
FFF13111983	DYT1	Filocamo	Male	nd	12	14-20
HF48	DYT1	Breakefield	Female	32	4	8-20

Abbreviation: nt, not determined; HF, human fibroblast; Healthy, apparently healthy controls; DYT1, primary human fibroblast cells from DYT1 dystonia patients; Breakefield, gift from Dr. Xandra Breakefield DYT1 fibroblast collection (Massachusetts General Hospital & Harvard Medical School, IRB no.: 2007P001632); Filocamo, gift from Dr. Mirella Filocamo (Biobank from Patients affected by Genetic Diseases, L'Istituto Giannina Gaslini, Genoa, Italy), member of the Telethon Network of Genetic Biobanks (project no.: GTB12001); and Coriell, purchased from Coriell Cell Repository (Camden, NJ, USA) (http://locus.umdnj.edu/ccr/).
